# Dietary supplementation with inulin improves burn-induced skeletal muscle atrophy by regulating gut microbiota disorders

**DOI:** 10.1038/s41598-024-52066-8

**Published:** 2024-01-28

**Authors:** Shan Gao, Xiaoshuai Zhao, Yan Leng, Zhongyuan Xia

**Affiliations:** Department of Anaesthesiology, Renmin Hospital of Wuhan University, Wuhan, Hubei China

**Keywords:** Nutrition, Malnutrition, Trauma, Microbiology techniques

## Abstract

Inulin, as a prebiotic, could modulate the gut microbiota. Burn injury leads to gut microbiota disorders and skeletal muscle catabolism. Therefore, whether inulin can improve burn-induced muscle atrophy by regulating microbiota disorders remains unknown. This study aimed to clarify that inulin intake alleviates gut microbiota disorders and skeletal muscle atrophy in burned rats. Rats were divided into the sham group, burn group, prebiotic inulin intervention group, and pseudo-aseptic validation group. A 30% total body surface area (TBSA) third-degree burn wound on dorsal skin was evaluated in all groups except the sham group. Animals in the intervention group received 7 g/L inulin. Animals in the validation group received antibiotic cocktail and inulin treatment. In our study inulin intervention could significantly alleviate the burn-induced skeletal muscle mass decrease and skeletal myoblast cell apoptosis. Inulin intake increased the abundances of Firmicutes and Actinobacteria but decreased the abundance of Proteobacteria. The biosynthesis of amino acids was the most meaningful metabolic pathway distinguishing the inulin intervention group from the burn group, and further mechanistic studies have shown that inulin can promote the phosphorylation of the myogenesis-related proteins PI3K, AKT and P70S6K and activate PI3K/AKT signaling for protein synthesis. In conclusion, inulin alleviated burn induced muscle atrophy through PI3K/AKT signaling and regulated gut microbiota dysbiosis.

## Introduction

Burn trauma results in severe and persistent skeletal muscle atrophy and gut microbiota disorders. Skeletal muscle wasting is considered to be a central factor in the pathophysiology of burn status, which can be prolonged due to the systemic inflammatory response, stress response, and restriction of physical activity^1^, thus prolonging burn healing. This persistent inflammation and cachexia is common in many trauma, critical and chronic conditions but is more severe and persistent in burn patients^2^.

In addition to causing muscle hypermetabolism, burns cause changes in the composition and diversity of the gut microbiota. A study using time series showed that the composition of the gut microbiota was significantly modified and gradually remodeled after severe burn trauma, with an initial decrease in biodiversity followed by a return to normal levels^3^. In burn patients, a decrease in the abundance of probiotic genera was observed, in addition to an increase in the abundance of opportunistic pathogens^4^. Studies in animal models of burns have confirmed that the abundance of bacterial components is affected to varying degrees, with severe intestinal barrier dysfunction and microbial community dysbiosis^5,6^. However, the link between burn-induced disruption of the gut microbiota and burn-induced muscle atrophy remains poorly understood.

Although muscle atrophy results in negative nitrogen balance and cachexia, thereby prolonging burn healing, it may also affect quality of life and increase mortality and is exacerbated by multiple surgeries with forced bedrest and immobility^1^. The focus of early rehabilitation for burn patients remains on wound healing and preventing hypertrophic scars and is inclined towards prophylactic measures to prevent muscle atrophy^7^. Therefore, in-depth understanding of the underlying processes of the gut microbiota and muscle atrophy and the beneficial effects of modulating the gut microbiota is clinically important for improving skeletal muscle atrophy after burns.

The gut microbiota constitutes the community of microbes present within the gastrointestinal tract^8^. This microbial ecosystem plays an important role in the host, facilitating the metabolism of dietary nutrients, synthesizing micronutrients and cofactors, modulating the mucosal immune system, and influencing energy balance^9^. Inulin, a prebiotic fibre, affects the levels of *Bacteroidetes* and *Bifidobacteria* in the gut microbiota, which can improve host gene expression and metabolism in animal models^10,11^. Dietary supplementation inulin also attenuated age-related muscle loss in aged rats^12^. Inulin could exert beneficial anti-inflammatory effects through short-chain fatty acid metabolites fermented by gut microbiota^13^, and also alleviate intestinal inflammation by modulating the intestinal barrier and gut microbiota^14^. However, studies on the improvement in the gut microbiota of burn patients with inulin have not been reported.

This study aimed to investigate the effect of supplementation with inulin to modify gut microbiota dysbiosis in burned rats and to assess its effect on muscle atrophy. We revealed that inulin ameliorates burn-induced gut microbiota disorders and alleviates skeletal muscle wasting by improving protein biosynthesis.

## Results

### Inulin treatment attenuates muscle wasting in burned rats

**Figure 1:**
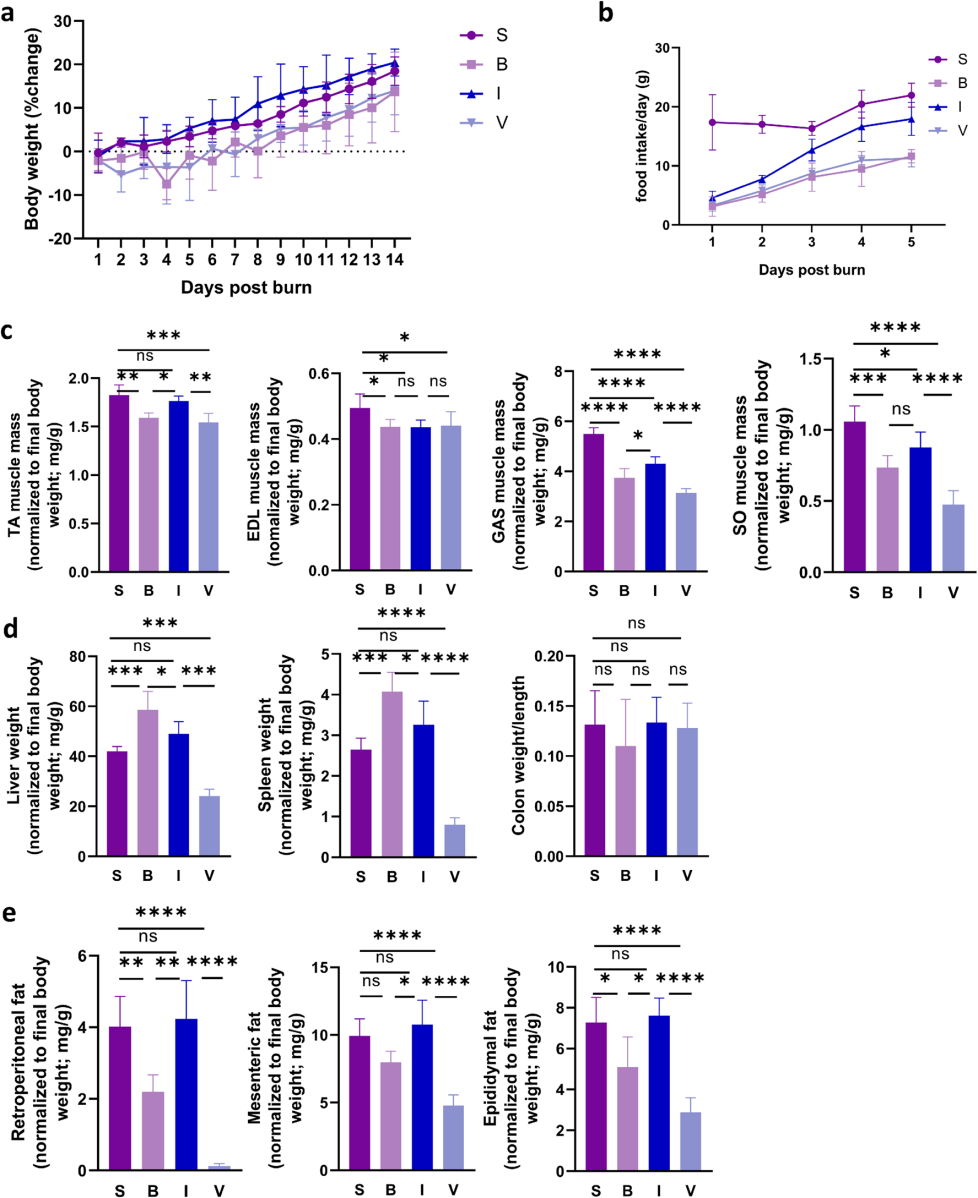
General characteristics with disproportionate muscle atrophy in the 30% total body surface area (TBSA) burn model. (**a**) Body weight changes. (**b**) Daily food intake of rats in different groups after burning. (**c**) Relative weights of the TA, EDL, GAS and SO muscles. (**d**) Relative weights of the liver and spleen and the ratio of colon weight to length. (**e**) Relative weights of retroperitoneal fat, mesenteric fat, and epididymal fat. All data are represented as the mean ± SEM and were analysed by T test or one-way ANOVA. *p < 0.05, **p < 0.01, ***p < 0.001, ****p < 0.0001. *S* sham, *B* burn, *I* intervention, *V* validation, *TA* tibialis anterior, *EDL* extensor digitorum longus, *GAS* gastrocnemius, *SO* soleus, *SEM* standard error of the mean.

To assess whether inulin could attenuate muscle atrophy after severe burn trauma, we examined animal weight, daily food intake, wet muscle weight, internal organs and fat weight. As expected, the percentage change in the body weight of rats in the burn (B) group and validation (V) group continued to decrease until 6 days after burn injury, after which the percentage change in body weight showed an upward trend. In addition, group B showed the most pronounced weight loss on the fourth day after burn injury, with a weight loss of approximately 7.28% relative to baseline weight. In contrast, with inulin treatment, the intervention (I) group showed a negative weight change for 4 days after the burn, and then an increasing trend was observed with the percentage of weight change decreasing most significantly on the first day after the burn. Moreover, group I showed only a 2.94% decrease in body weight on the second day after the burn, which was a difference that was significant compared to groups B and V. The rats in sham (S) group showed an increasing trend in the percentage change in body weight after sham burn (Fig. 1a).

Daily food intake in groups B, I and V all showed an increasing trend. On the first day postburn, the average food intake in group B was only approximately 3 g. During the first two days, there was no significant difference in the average daily food intake between groups B, I and V. However, the mean daily food intake was higher in group I than in groups B and V and lower in group I than in group S (Fig. 1b).

Burn-induced organomegaly increased total body weight and masked the severity of lean body mass loss. Therefore, we also weighed the muscles, organs, and fats of the rats. The change in wet muscle weight is shown in Fig. 1c. Compared to group S, the wet muscle weight of the tibialis anterior (TA), extensor digitorum longus (EDL), gastrocnemius (GAS), soleus (SO) was lower in group B. After the dietary intervention, the reduction in wet TA and GAS weights was less severe in group I compared to group B. Group V, which lacked gut microbiota, showed a decrease in TA, GAS and SO muscle weight compared to group I, suggesting that the gut microbiota is essential for maintaining muscle mass.

Burn-induced organomegaly, such as in the liver and spleen, is presented in Fig. 1d, and the weights of the liver and spleen were significantly higher in group B than in group S. The weights of the liver and spleen were decreased in group I compared with group B. We also analysed the relative weights of retroperitoneal fat, mesenteric fat, and epididymal fat, and burn injury resulted in significant lipolysis. These factors improved significantly in response to inulin supplementation in group I. There was no significant difference in the colon weight/length ratio in the four groups throughout the experimental period (Fig. 1e).

### Inulin treatment attenuates burn-induced muscle atrophy

**Figure 2:**
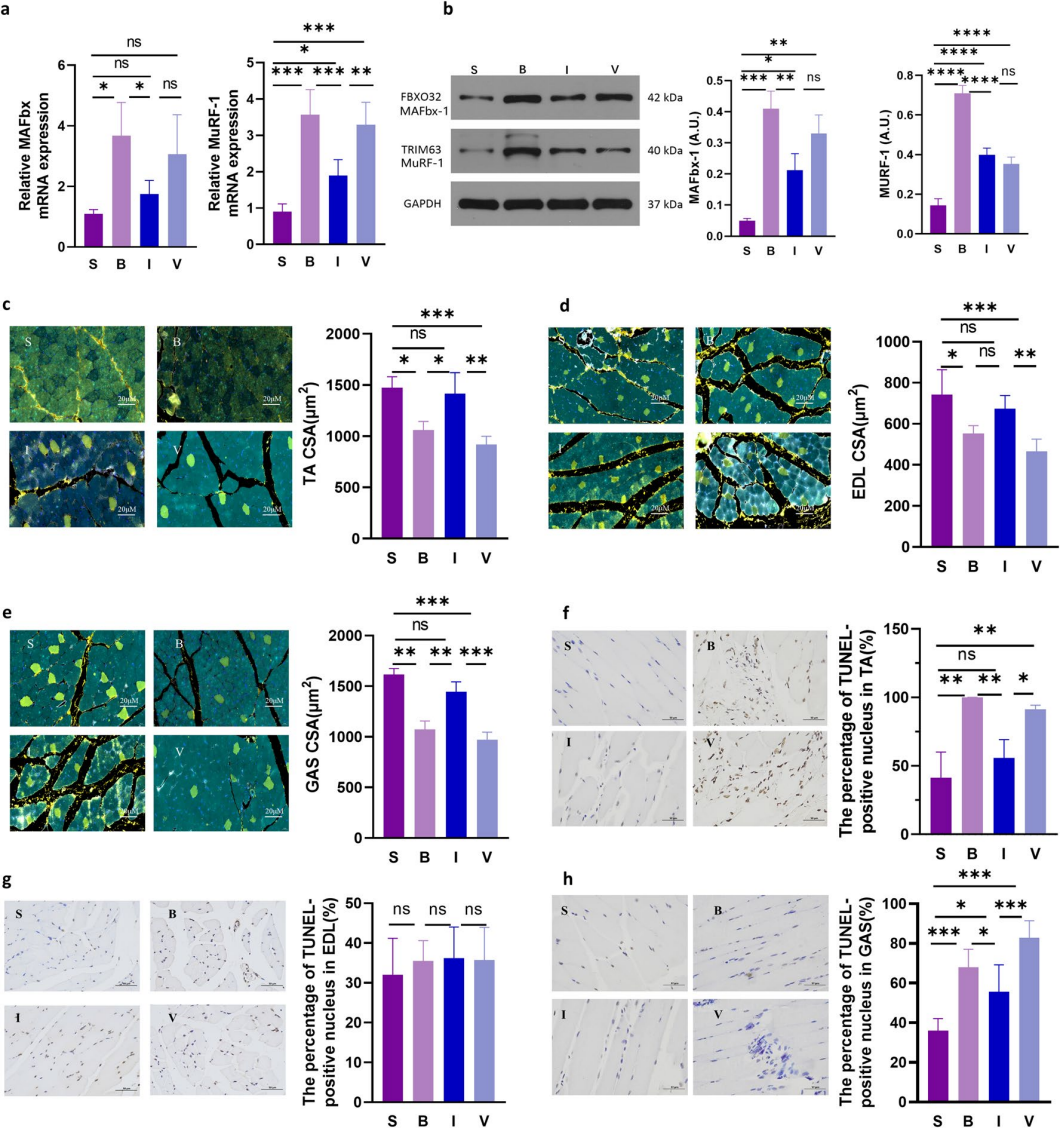
Muscle atrophy and muscle wasting in the 30% total body surface area (TBSA) burn model. (**a, b**) PCR measurement of MAFbx and MuRF1 mRNA levels and Western blot analysis of MAFbx and MuRF1 proteins in the TA at 4 days after burn. (**c, d, e**) Immunofluorescence staining of TA, EDL and GAS sections in four groups at 4 days after burn and variation in myofiber cross-sectional area (CSA), respectively. Five representative views were randomly selected to calculate statistical significance (here muscle cryosections immunostained for DPAI(blue), laminin (white), MyHC I (yellow), MyHC IIa (cyan), and MyHC IIb (orange)). Scale bar = 20 μm. (**f, g, h**) The proapoptotic effects of burn trauma on TA, EDL, GAS cells were assessed by TUNEL staining. Nuclei were stained blue with haematoxylin, and TUNEL-positive apoptotic nuclei were stained brown‒yellow with DAB reagent. Scale bar = 50 μm. The percentage of TUNEL-positive nuclei in the TA, EDL and GAS; three representative views were randomly selected to calculate statistical significance. All data are represented as the mean ± SEM and were analysed by t test or one-way ANOVA. *p < 0.05, **p < 0.01, ***p < 0.001, ****p < 0.0001. *S* sham, *B* burn, *I* intervention, *V* validation, *TA* tibialis anterior, *EDL* extensor digitorum longus, *MAFbx* muscle atrophy F-box, *MuRF1* muscle-specific ring finger protein 1, *SEM* standard error of the mean.

To investigate the contributions of skeletal muscle catabolic processes, we measured the gene and protein expression of the skeletal muscle-specific E3 ubiquitin-protein ligases Muscle atrophy-related F-box (MAFbx) and Muscle atrophy-related F-box (MuRF1) in the TA (Fig. 2a). We observed that total MAFbx mRNA levels were approximately threefold higher in groups B and V than in group S. Similarly, we also observed that total MuRF1 mRNA levels were more than threefold higher in groups B and V than in group S. Consistent with previous reports of increased expression of the RNA encoding MAFbx^15^ and MuRF1^16^, our study also observed enhanced RNA expression of MAFbx and MuRF1 in animals with skeletal muscle atrophy. This overexpression coincided with significant TA muscle atrophy observed at 4 days post-burn. However, the MAFbx and MuRF1 mRNA levels in group I, which were lower than those in group B, indicated that inulin alleviated muscle wasting in the burned rats.

In the TA muscle, we also observed markedly elevated protein expression of MAFbx and MuRF1 in group B (Fig. 2b). In contrast, significantly lower protein expression of MAFbx and MuRF1 in group I was observed, indicating that inulin reduced protein expression and activity in the ubiquitin‒proteasome system signaling pathway to a similar extent. The lower MAFbx and MuRF1 protein expression in group V than in group B was not consistent with a decrease in muscle mass, suggesting that the protein synthesis pathway may be inhibited in the absence of gut microbes known to regulate muscle mass. These results suggest that muscle atrophy postburn is associated with increased activity of MAFbx and MuRF1, and inulin treatment leads to inhibition of the UPS involved in protein degradation.

Immunofluorescent (IF) staining of TA, EDL and GAS sections was used to determine muscle fibre atrophy in the four groups (Fig. 2c–e). Consistent with the overall reduction in muscle mass, myofiber cross-sectional area (CSA) was significantly reduced in groups B and V. Indeed, in our model of burn-induced muscle atrophy, inulin treatment attenuated the CSA of the TA and GAS 4 days after burn injury. In group V, the amelioration of muscle atrophy by inulin treatment was inhibited by antibiotics. These results suggest that inulin attenuates muscle atrophy in severely burned rats (scale bar = 20 μm).

In addition to muscle protein degradation, burn trauma can trigger muscle wasting through apoptosis, which occurs in muscle that is both proximal and distal to the burn^17^. To examine burn-induced skeletal muscle apoptosis, we performed Terminal deoxynucleotidyl transferase-mediated dUTP biotin nick-end labelling (TUNEL) staining in the TA, EDL and GAS (Fig. 2f–h). In group S, no significant changes in skeletal muscle apoptosis were observed in the TA and GAS; however, significant skeletal muscle apoptosis was observed in groups B and V. Moreover, apoptosis was observed in group I but was significantly less than in group B. While the number of TUNEL-positive nuclei in groups B and V was significantly higher than in the S group, the percentage of TUNEL-positive nuclei decreased after inulin treatment. The protective effect of inulin was reversed by antibiotics, and the number of apoptotic nuclei was higher in group V than in group I. TUNEL staining of the EDL showed no statistically significant difference in the number of TUNEL-positive nuclei among the four groups (scale bar = 50 μm).

### Inulin treatment altered cytokine and intestinal tight junction (TJ) protein expression in burned rats

**Figure 3:**
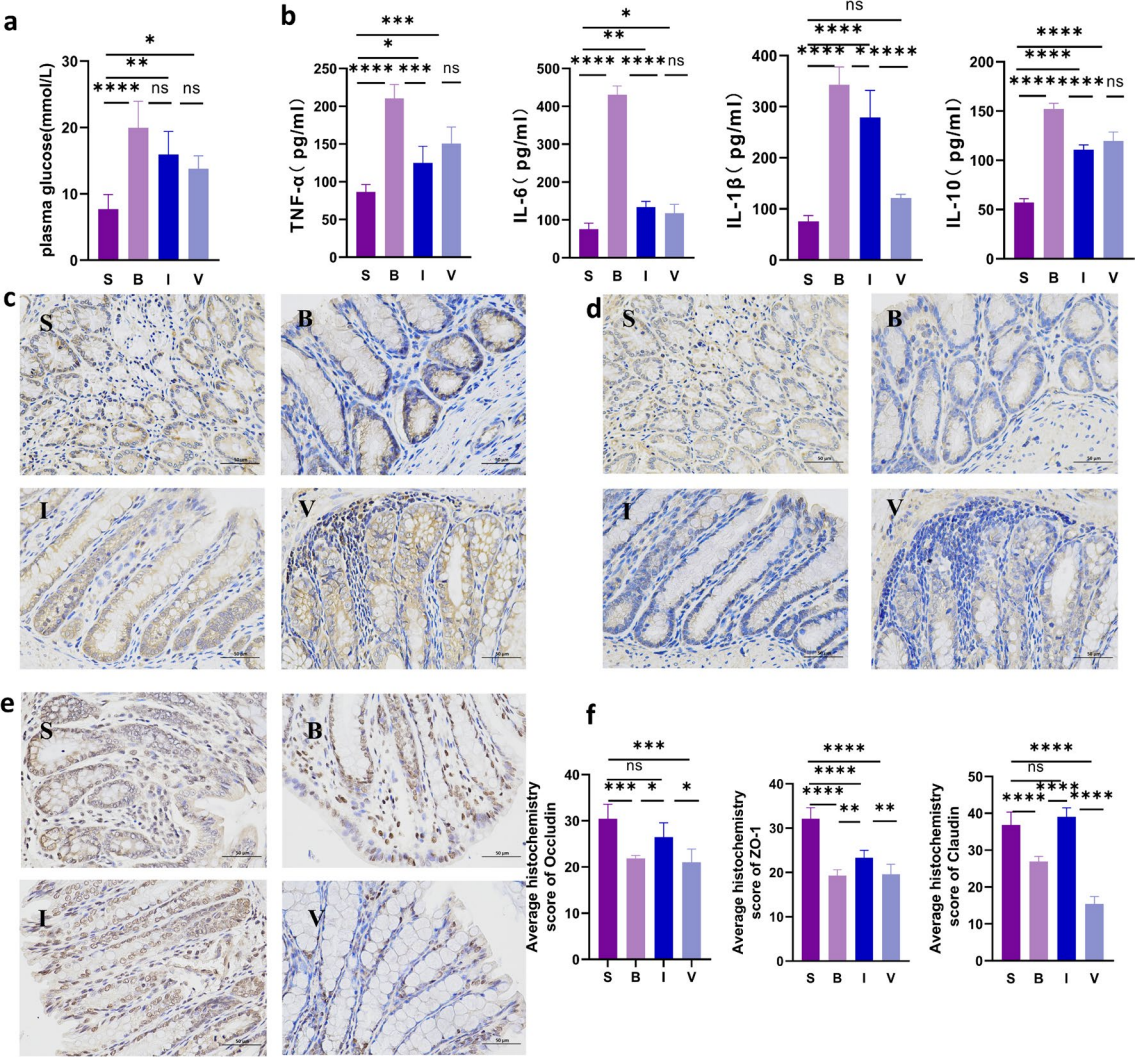
Plasma glucose, serum cytokine levels and colon tight junction protein expression in the four groups. (**a**) Plasma glucose level. (**b**) Serum cytokine levels. (**c-e**) IH staining for occludin, ZO-1, and claudin expression using the DAB visualization method. (**f**). H-score of occludin, ZO-1, and claudin staining. (n = 5 slides per condition; mean ± SEM) *p < 0.05, **p < 0.01, ***p < 0.001, ****p < 0.0001. *S* sham, *B* burn, *I* intervention, *V* validation, *ZO-1* zonula occludens-1, *IL-1β* interleukin-1, *IL-6* interleukin-6, *IL-10* interleukin-10, *TNF-α* tumor necrosis factor-α, *SEM* standard error of the mean.

Detection of plasma glucose and cytokine levels in serum found that inulin reduced the level of inflammatory factors (Fig. 3a,b). The expression of the TJ proteins occludin, zonula occludens-1 (ZO-1) and claudin in the colons of rats on the fourth day after burn injury was detected by Immunohistochemical (IH) staining. In this study, severe burns resulted in a significant decrease in intestinal TJ protein expression compared to that in the sham group (Fig. 3c–f). The histochemistry score (H-score) of occludin was significantly reduced in the three burn treatment groups compared to group S. Occludin expression showed an increasing trend in group I compared to groups B and V, while the administration of antibiotics eliminated inulin-induced effects of the gut microbiota and occludin expression in the intestine. The H-score of ZO-1 was significantly higher in group S than in the other three groups (*P* < 0.001), while it was higher in group I, which was fed inulin, compared to groups B and V (*P* < 0.05). In antibiotic-treated rats (group V), the effect of inulin, which increases the expression of ZO-1 during burn trauma, was inhibited. The same trend was also observed for claudin expression in the four groups (scale bar = 50 μm).

### Inulin treatment altered gut microbiota dysbiosis

**Figure 4:**
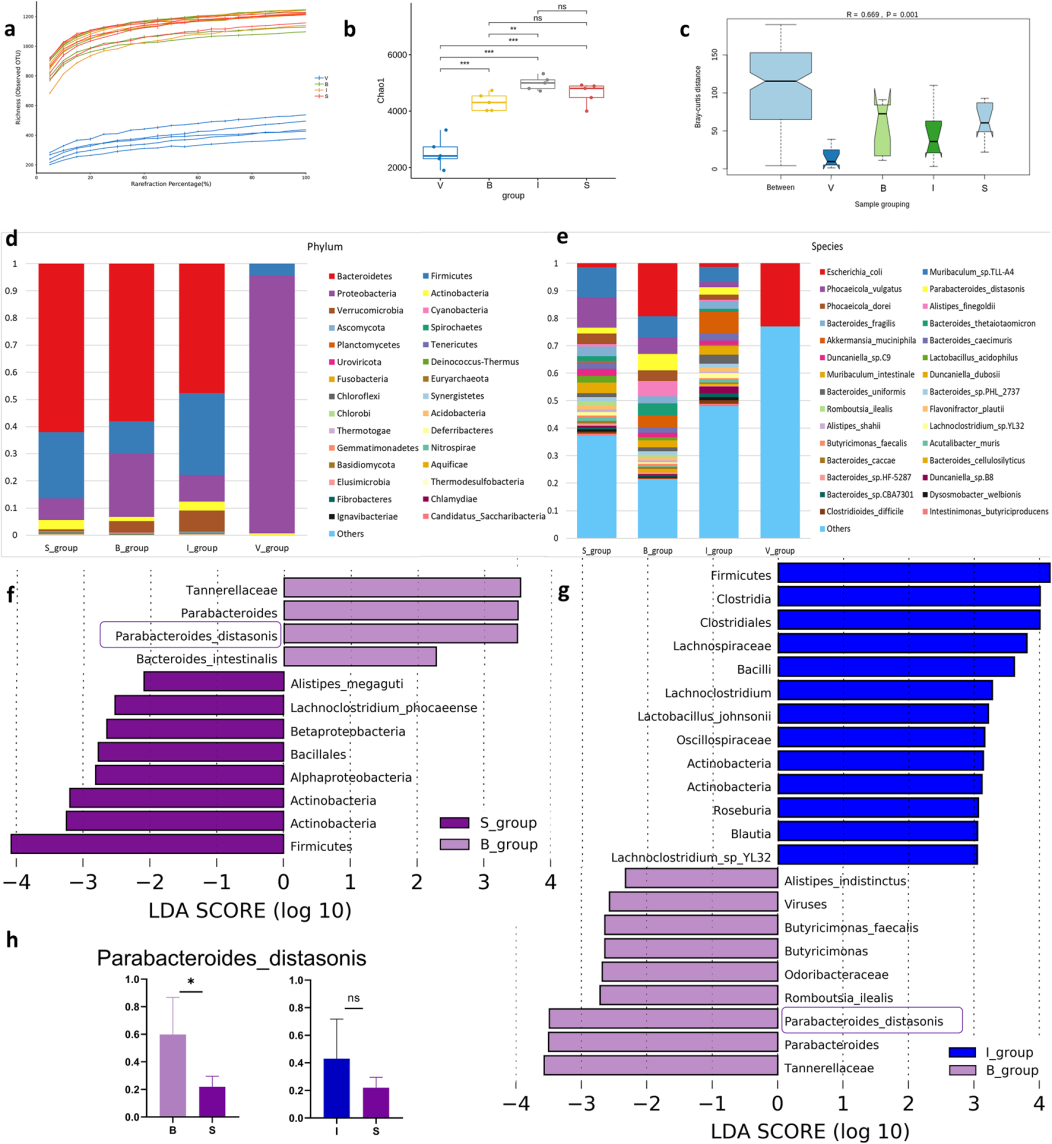
Taxonomic analysis of the gut microbiota in different treatment groups. (**a**) Rarefaction curve analysis in four groups. (**b**) Alpha diversity of the four cohorts at the species level, measured in terms of the Chao1 index. (**c)** Beta diversity of the four cohorts at the species level. (**d**) Bar plot of taxonomy analysis between four groups in taxonomic levels of phylum. (**e**) Bar plot of taxonomy analysis between four groups in taxonomic levels of species. The horizontal axis is the group, and the vertical axis is the relative abundance of certain species. (**f, g**) LEfSe analysis LDA value information at each taxonomic level between different groups. The ordinate shows species with significant differences in LDA values greater than the present value (2.0) in different groups, that is, biomarkers with significant differences. The length of the bar in the chart represents the impact of significantly different species. (**h)** *P. distasonis* species relative abundance in different groups. The *P* value was determined by a two-tailed Wilcoxon rank-sum test, and data are presented as the means ± SEMs; * *p* < 0.05. *S* sham, *B* burn, *I* intervention, *V* validation, *LEfSe* linear discriminant analysis effect size, *LDA* linear regression analysis, *P. distasonis Parabacteroides_distasonis, SEM* standard error of the mean.

Since we demonstrated that inulin affected muscle mass in burned rats, we hypothesized that inulin might improve burn-induced muscle wasting through the gut–muscle axis. Therefore, we investigated the effect of inulin on the gut microbiota. Rarefaction analysis was performed to determine whether the sequencing adequately captured the genetic diversity of the gut microbiota. We could see that the curves for all samples were close to saturation, which indicates that the sequencing depth and sample size were reasonably sufficient to capture most of the genetic diversity (Fig. 4a).

The Chao1 diversity index analysis showed that inulin supplementation increased the alpha diversity of the microbiota in burned rats compared with that in group B (Fig. 4b). The results of the Bray‒Curtis distance analysis revealed that the beta diversities were different among the four groups (Fig. 4c).

The taxonomic assignment for the metagenomic data was carried out and shown using a bar plot. The relative abundance of gut microbes was calculated by summing the abundance of genes. The relative abundances of the gut microbes in different groups at the phylum level and species level are shown in Fig. 4d,e. In the sham group, the relative abundances of *Bacteroidetes, Firmicutes, Proteobacteria,* and *Actinobacteria* were 62%, 24%, 7.8% and 3.6%, respectively. In the burn group, the gut microbiota was changed compared with that in the sham group, the relative abundances of *Bacteroidetes, Firmicutes* and *Actinobacteria* were decreased (58%, 12% and 1.4%), and the relative abundance of *Proteobacteria* was increased (23%). The *Firmicutes/Bacteroidetes* ratio *(F/B* ratio) was decreased. Compared to the burn group, inulin increased the relative abundance of *Firmicutes* and *Actinobacteria* (30% and 3.3%, respectively) in the intervention group and decreased the abundance of *Proteobacteria* (9.7%). We observed significant differences in the relative abundance of species across treatment groups (Fig. 4e). These results indicate the differences in the gut microbial communities of rats in different treatment groups.

To further explore features of the gut microbial community in burned rats, we analyzed the taxonomic differences between different groups using Linear discriminant analysis effect size (LEfSe). At the genus and species levels, *Parabacteroides* and *P. distasonis* were the most significantly different genera and species in group B compared to group S (Fig. 4f). The *Parabacteroides* genus has been reported to have both beneficial and pathogenic effects on human health. Individuals with burn treatment showed a significant increase in the relative abundance of *P. distasonis* compared with rats in the sham group, and the relative abundance of *P. distasonis* was decreased in group I, which indicated that inulin could improve gut dysbiosis (Fig. 4g). Relative abundance histograms were generated based on species with intergroup differences detected in the LEfSe results; at the species level, the abundance of *P. distasonis* was higher in group B than in group S, while there was no difference in the abundance of *P. distasonis* between group I and group S (Fig. 4h). Therefore, *P. distasonis* was the biomarker species identified in group B.

### Inulin treatment altered the gut microbiota functional annotation

We performed functional analysis of the gut microbiota between the burn and sham groups. For nonredundant genes, the BLASTP function of Diamond is generally used for functional annotation. Then, the results were compared with the Kyoto Encyclopedia of Genes and Genomes (KEGG) database. All the KOs (KEGG orthology) involved in a particular pathway were statistically analyzed through the Reporter Score.

**Figure 5:**
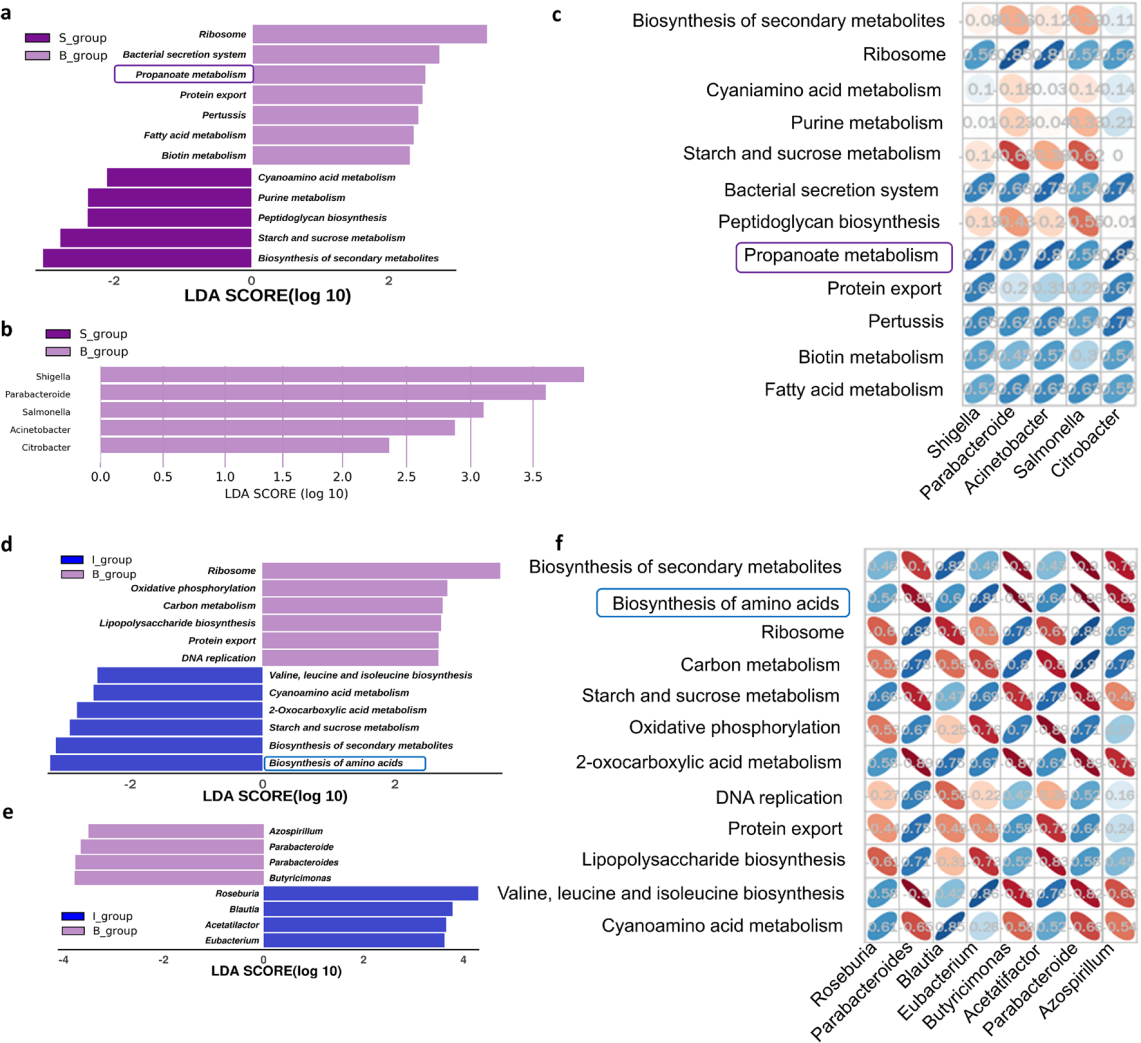
Functional annotation of the gut microbiota in different treatment groups. (**a**) Annotation boxplot of differential function between group B and group S. (**b**) KEGG pathway analysis of differentially expressed genes between group B and group S. (**c**) Ellipse heatmap of differentially expressed genes and differential pathway relationships. (**d**) Annotation boxplot of differential function between group B and group I. (**e**) KEGG pathway analysis of differentially expressed genes between group B and group I. (**f**) Ellipse heatmap of differentially expressed genes and differential pathway relationships. a t test of two independent samples p < 0.05 as differential species, LEfSe analysis LDA > 2 as differential pathways. *S* sham, *B* burn, *I* intervention, *V* validation, *LEfSe* linear discriminant analysis effect size, *LDA* linear regression analysis.

Then, we further analyzed the different functions between different groups using LEfSe. The pathways that were significantly enriched in group B were ribosome, bacterial secretion system, propanoate metabolism, protein export, pertussis, fatty acid metabolism, biotin metabolism and base excision repair. The pathways that were significantly enriched in group S were biosynthesis of secondary metabolites, starch and sucrose metabolism, peptidoglycan biosynthesis, purine metabolism and cyanoamino acid metabolism (Fig. 5a). The bacterial ribosome is the site where genetic information is translated into proteins. The bacterial secretion system is associated with bacterial virulence. Thus, propionate metabolism is the most meaningful metabolic pathway that distinguishes group B from group S. At the gene level, differential genes between groups were obtained using LEfSe analysis, and the top five differential genes in group B are shown here for *Parabacteroides, Shigella, Acinetobacter, Salmonella,* and *Citrobacter* (Fig. 5b). Then, we analyzed the correlation between differentially expressed genes and differentially enriched metabolic pathways. There was a significant positive correlation between the levels of the differential strains that were significantly enriched in group B and the propanoate metabolism pathway (Fig. 5c).

LEfSe analysis of functional annotation of group B and group I revealed enhanced expression of molecules involved in ribosome, oxidative phosphorylation, carbon metabolism, lipopolysaccharide biosynthesis, protein export, and DNA replication pathways in group B (Fig. 5d). Oxidative phosphorylation provides ATP for hypermetabolism in burns, and lipopolysaccharide biosynthesis and entry into systemic circulation cause endotoxaemia. At the gene level, after the differential genes between groups were identified using LEfSe analysis (Fig. 5e), the differential KEGG pathways were correlated in the differential genera (Fig. 5f). Biosynthesis of amino acids is the most meaningful metabolic pathway in group I, which may be related to skeletal muscle protein synthesis, so we subsequently performed WB of the expression of key proteins in the protein biosynthesis signaling pathway.

### Inulin promotes skeletal muscle protein synthesis via Phosphatidylinositol 3-kinase/protein kinase B (PI3K/AKT) signaling

**Figure 6:**
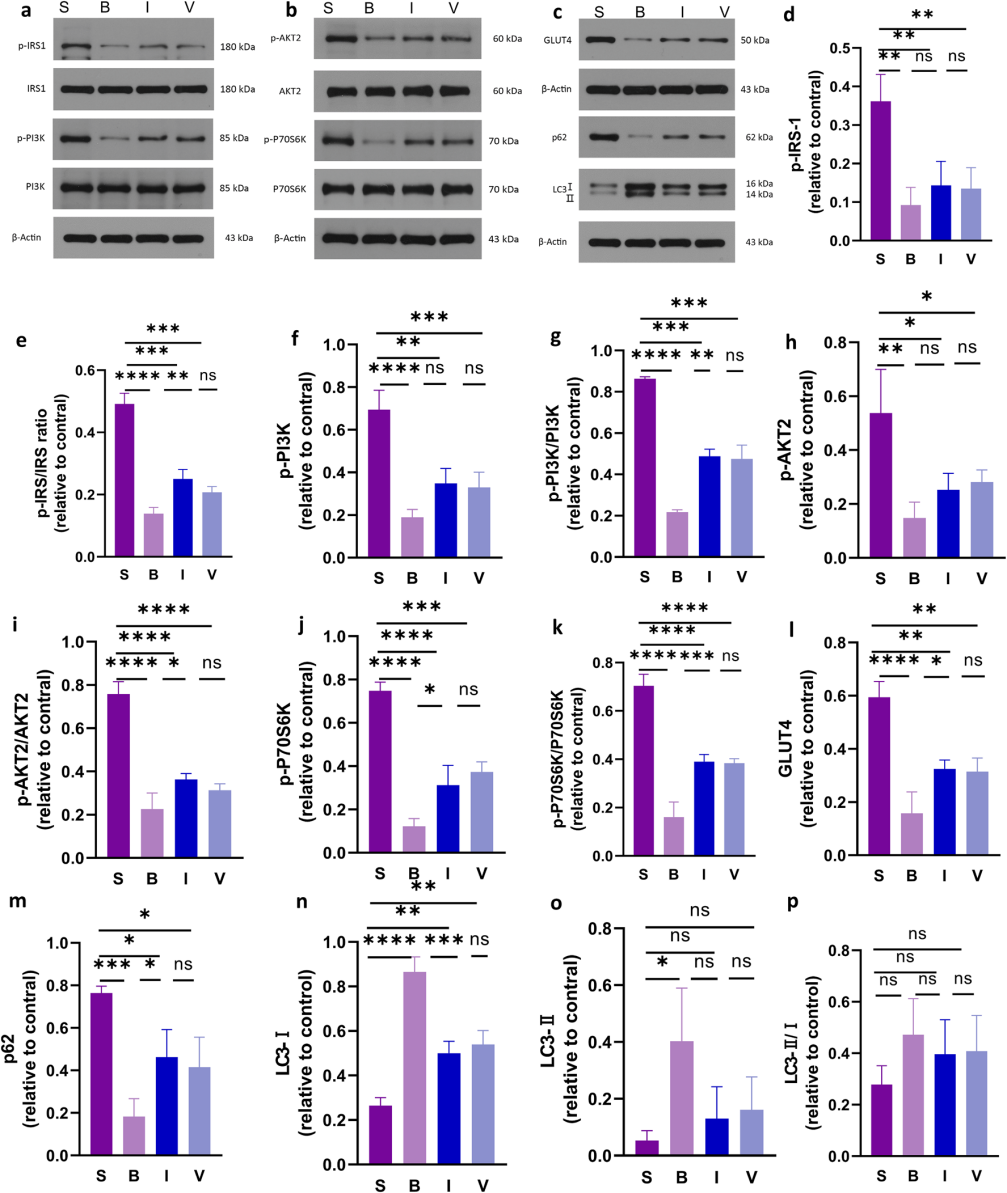
Inulin treatment attenuates protein synthesis in burned rats. (**a-p**) Changes in PI3K/AKT signaling and glucose transporter and autophagy-related protein expression in different groups analyzed by Western blotting. One-way ANOVA was applied to test the statistical significance. Data are expressed as the means ± SEMs (* *P* < 0.05, *p < 0.01, ***p < 0.001, ****p < 0.001). *S* sham, *B* burn, *I* intervention, *V* validation, *IRS* insulin receptor substrate, *P13K* phosphatidylinositol 3-kinase, *AKT* protein kinase B, *S6K* S6 kinase, *GLUT4* glucose transporter type 4, *SEM* standard error of the mean.

Insulin mediates PI3K/AKT signaling and plays an important role in protein synthesis in skeletal muscle cells. We next detected the effect of inulin-induced anabolic cellular signaling pathways in TA muscle (Fig. 6a–c). Phosphorylation of IRS, PI3K, Akt and P70S6K is inhibited in burned rats. Inulin induces phosphorylation of IRS, PI3K, AKT and P70S6K (Fig. 6d–k). Glucose transporter GLUT4 was also upregulated after inulin treatment, suggesting that inulin treatment could have facilitated glucose uptake by skeletal muscle (Fig. 6l). In the acute phase, burn injury induces upregulation of autophagy and conversion of LC3I to LC3II, and the feedback increases the expression of LC3. In our results, the autophagic structural protein LC3II levels increased and the selective autophagy junction protein p62 levels decreased in group B, and inulin improved excessive autophagy by decreasing LC3II levels and increasing p62 levels (Fig. 6m–p). All bands are visible in the supplementary file. Taken together, these results suggest that inulin is a regulator of protein synthesis that maintains high Akt activity and protein synthesis and reduces excessive autophagy in skeletal muscle.

## Discussion

Severe burns result in profound and permanent disorders of the gut microbiota^18^. Current strategies for the nutritional and metabolic support of burn patients are limited, and few effective strategies are available to reduce long-term muscle wasting after burn injury. Therefore, our study is the first to provide a novel and effective strategy for providing prebiotics via the gut-muscle axis to ameliorate muscle atrophy in burn injury.

Emerging evidence suggests the benefits of dietary fiber in the regulation of the gut microbiome to improve glucose and lipid metabolism^19,20^, influence cardiometabolic outcomes^9,21^, and modulate immune status^22^ and body health^23^ due to the important plasticity of the gut microbiota^24^. However, whether dietary fiber could ameliorate skeletal muscle atrophy in burned rats by regulating the gut microbiota has not yet been studied. Consistent with previous research, our study also revealed that burn injury resulted in persistent protein breakdown and muscle wasting, especially myofibrillar protein breakdown, and these changes were substantially more obvious in fast-twitch muscles (TA and GAS)^25,26^. In contrast, dietary supplementation with inulin effectively alleviated the weight loss and muscle mass reduction in burned rats, and the reduction in myofiber CSA and the degree of skeletal muscle cell apoptosis were significantly improved. In addition to dietary fiber, dietary addition of proteins such as soybean protein-derived peptides improved the expression of burn-induced inflammatory markers and atrophy-associated autophagy signaling pathways in burned rats^27^. Protein supplementation significantly increased lean body mass, but not muscle strength in burn patients^28^. Isolated soy protein and flaxseed oil also reduced muscle catabolism and increased body weight in burn patients^29^. Dietary supplementation with inulin was also observed to improve weight loss and skeletal muscle atrophy in burned rats, probably due to the modulatory effect of inulin on the gut microbiota, which thereby improved metabolism.

The gut microbiota is important for the maintenance of skeletal muscle mass and function. Burns reduce the *F/B* ratio, and *Firmicutes* and *Bacteroidetes* are the dominant phyla of the gut microbiota. Changes in the *F/B* ratio are associated with metabolic disease and insulin resistance and affect nutrient uptake. Increased *F/B* ratios have been observed in individuals with autism^30^, inflammatory bowel disease and obesity^31^, which are all associated with several inflammatory conditions. In contrast, the *F/B* ratio decreases in patients with cachexia (colorectal, breast, and lung cancer)^32–34^ and noncancerous cachexia, manifested by chronic muscle wasting, chronic kidney disease or liver disease^35^. The development of age-related sarcopenia is also associated with dysbiosis of the gut microbiota^36^. The relative abundance of the phylum *Firmicutes* was decreased in older adults with sarcopenia, and the dominant microbiota at the genus level were *Dialister, Lactobacillus* and *Ruminococcus*^37^.

Consistent with the increased relative abundance of *Proteobacteria* as a result of burn injury, *Proteobacteria* are frequently associated with dysbiosis in gut microbial communities that include an increase in endotoxin biosynthesis (notably from *Proteobacteria*) and an increase in gut permeability^38^. Many studies have reported that *Proteobacteria* is a biomarker phylum for inflammatory bowel disease, obesity, diabetes, and other metabolic diseases^39^. In particular, a relative increase in the levels of *Proteobacteria* was observed in burn-induced gut microbiota dysbiosis, with a concomitant reduction in *Firmicutes* levels. *Proteobacterial* colonization disrupts mucosal barrier function and leads to the development of intestinal inflammation, resulting in changes in small intestine morphology (reduced surface area, increased permeability, and apoptosis of epithelial cells) and ultimately impairments in intestinal absorption^40^.

In this study, the addition of prebiotic inulin resulted in an increase in the abundance of *Firmicutes*, a decrease in the abundance of *Proteobacteria* and an increase in the *F/B* ratio in the gut microbiota and ameliorated skeletal muscle atrophy and myocyte apoptosis in burn-injured rats. In contrast, skeletal muscle atrophy was not alleviated by the dietary addition of inulin in burned rats treated with antibiotics, suggesting that inulin alleviates skeletal muscle atrophy by improving the intestinal microbiota disorder caused by burn injury. Inulin intake can regulate the composition and activity of the intestinal microbiota, and inulin can also be fermented by the microbiota to produce SCFAs to improve sensitivity to insulin and significantly improve muscle function and muscle mass in elderly individuals^41^. Burn trauma alters the expression of tight junctions and the composition of the gut microbiota, further impairing the intestinal barrier and increasing intestinal permeability, leading to pathological transfer of gut bacteria or endotoxins^42^. We found that inulin inhibited the increase in *Proteobacteria* levels and promoted the expression of tight junction proteins, such as claudin-1, occludin, and ZO-1. In addition, inulin also regulates skeletal muscle metabolism by promoting PI3K/AKT signaling pathway phosphorylation to promote protein synthesis and reduce burn-induced excessive autophagy.

*Parabacteroides distasonis* is a well-characterized biomarker of burns, and inulin supplementation could significantly reduce the overrepresentation of *P. distasonis*. Recent research in microbiological fields has found *P. distasonis* to be a highly controversial bacterium. Both in vitro and in vivo studies have revealed that *P. distasonis* displays anti-inflammatory effects and the ability to restore the epithelial barrier in a cell culture model and strengthen the intestinal barrier to attenuate intestinal inflammation in a mouse model of colitis^43^. A prebiotic diet increases the relative abundance of *P. distasonis*, improves sleep during repeated sleep interruptions and sleep resumption^44^, alters the fecal bile acid profile, and promotes the recovery and readjustment of sleep and circadian rhythms after circadian rhythm interruptions^45^. However, *P. distasonis* could induce depressive-like behavior by synthesizing neurotransmitters in a mouse with Crohn’s disease^46^ and exacerbate the symptoms of amyotrophic lateral sclerosis^47^.

We observed a significant enhancement in the metabolic pathway of propionate in burned rats, while showing a positive correlation with the elevated relative abundance of *Parabacteria, Shigella, Acinetobacter, Salmonella* and *Citrobacter*. One study reported that exposure to propionate increases propionylation, impairs the differentiation of primary human myotubes and murine myotubes, and promotes propionylation in the regulatory promotor regions of Myod^48^. Another clinical study found abnormal metabolic patterns in the muscle bioenergetic metabolism of patients with muscular dystrophy in the early or acute phase of the disease, with abnormally elevated propionate^49^. The accumulation of propionate and propionyl-CoA has been associated with the development of mitochondrial disorders. Propionyl-CoA accumulation suppresses the activity of mitochondrial respiration complexes, which implicates the involvement of protein propionylation^50^ . Genetic defects in the propionyl-CoA carboxylase gene result in patients with propionyl-CoA accumulation clinically presenting with symptoms associated with mitochondrial defects, characterized by decreased mitochondrial respiration^51^. However, the potential links between propionate and burn-induced muscle atrophy have never been studied.

## Conclusion

In conclusion, we have shown that burn-induced skeletal muscle atrophy is associated with specific alterations in the gut microbiota composition and propionate metabolism in rats. These insights represent a critical first step and highlight the need to assess whether interventions targeting the gut microbiota could be a strategy for treating burned skeletal muscle atrophy. Given the limited efficacy of current therapies and the considerable impact of skeletal muscle atrophy on the prognosis and quality of life of burn patients, innovative strategies to alleviate skeletal muscle atrophy are urgently needed.

## Methods

### Animal experiments

All animal experiments were performed with approval by the Animal Ethics Committee of Tongren Hospital, Wuhan University, and care of the animals was in accordance with the National Institutes of Health guidelines for animal treatment. Male Sprague‒Dawley rats that were 6–8 weeks old and weighed 180–210 g were purchased from Sanxia University, China, with certificate number SYXKI2020-0080. All rats were fed and given water ad libitum and kept in specific pathogen-free environments at a stable temperature (25 ± 3 °C) and relative humidity (50–60%) for a 12-h light–dark cycle. All rats were acclimated to living conditions for one week before burn models were established.

According to the guidelines, all animals were humanely cared for during all steps of the experiments. The experiment was divided into two parts: an acute experiment and a 15-day weight observation experiment. Body weight and food intake were recorded daily during the observation experiment. All rats were randomly divided into 4 groups: sham burn group (S group), burn group (B group), intervention group (I group) and validation group (V group). Animals in group I received a basic diet supplemented with 7 g/L inulin fiber (short-chain inulin, Vilof Co., Shanghai, China). Animals in group V, as antibiotic-treated rats, were used to establish burn models after two weeks of pretreatment with drinking water supplemented with antibiotics [ampicillin (1 g/L), metronidazole (1 g/L), neomycin (1 g/L) and vancomycin (0.5 g/L)] and then provided a basic diet supplemented with inulin fiber. The water intake was comparable for each treatment group.

### Burn injury model

A 30% TBSA third-degree burn wound was evaluated using the rat model. After anaesthesia, the experimental animals were shaved and disinfected using 70% ethanol. Afterwards, in all groups except group S, 30% of the TBSA of a third-degree scald wound was created using a scald device by placing it on the dorsal skin of rats for 20 s at 96 °C. The S group did not receive burn trauma but received anesthesia and had their backs shaved. Afterwards, the rats were resuscitated with an injection of prewarmed normal saline (5 mL per 100 g body weight) and housed individually in cages.

The rats were euthanized at 4 days following burn injury for acute studies. TA, EDL, GAS, SO, liver, spleen, colon, retroperitoneal fat, mesenteric fat, epididymal fat, blood and faecal samples were collected at 4 days after the burn. Blood was centrifuged at 12,000 rpm for 5 min at 4 °C, and the serum was collected. The muscle tissues, internal organs, and fat were removed and weighed at the time of harvest to obtain the wet muscle weight, and portions of the tissue were fixed in 4% paraformaldehyde for histological analysis. Fecal samples collected during the dietary intervention and samples collected at sacrifice were used for microbiome profiling. All the samples were stored at -80℃ until the assay was performed.

### IF staining for myofiber CSA analysis

We used immunofluorescence (IF) to stain the TA, EDL and GAS muscle fibers. Laminin (23498-1-AP, proteintech, Wuhan, China) was used to detect myofiber borders. To detect different myofibers, muscle Sects were incubated with antibodies specific to myosin heavy chain (MyHC) types I, IIa, and IIb (22280-1-AP, 55069-1-AP, and 20140-1-AP, respectively, Proteintech, Wuhan, China), unstained myofibers were judged as MyHC IIx expression. Alexa Fluor 546, CY3 and 568 secondary antibodies were used to detect MyHC types I, IIa, and IIb, respectively^52,53^. For each section, the average CSA of myofibers are presented as the total area of myofibers/total number of myofibers in 5 images (field of view).

### IH staining

The colon tissues were fixed in 4% paraformaldehyde, embedded in paraffin, dewaxed in xylene and rehydrated in gradient alcohols. The tissue sections were retrieved in citrate buffer (pH 6.0) and incubated with primary antibodies (rabbit anti-occludin, 1:500, GB111401, Servicebio; rabbit anti- zonula occludens-1, 1:200, A0659, Abclonal; rabbit anti-claudin, 1:500, GB11032, Servicebio) overnight at 4 °C. Next, these sections were incubated with horseradish peroxidase (HRP)-conjugated goat anti-rabbit IgG (1:200, GB23303, Servicebio, China) at 25 °C for 2 h. After washing in PBS, the sections were counterstained with haematoxylin for 3 min, dehydrated and mounted. For quantification, five fields were randomly selected for each sample. The expression of occludin, ZO-1 or claudin protein was quantified by the histochemistry score (H-score) by selecting five regions on each slice. The average H-score values from the five regions were statistically analysed to determine the overall percentage of occludin, ZO-1 or claudin protein-positive area across the four groups. H-score = ∑ (pi × i) = (percentage of weak intensity × 1) + (percentage of moderate intensity × 2) + (percentage of strong intensity × 3)^54,55^.

### TUNEL staining

Paraffin sections were made from 4% paraformaldehyde-fixed tissue. After the TA and EDL sections were washed and dewaxed, they were repaired with Proteinase K solution, and slides were rinsed twice in PBS and air dried. TUNEL reaction mixture (recombinant TdT enzyme, biotin-dUTP labelling mix, equilibration buffer) was then added to the specimens before the membrane was sealed and incubated. An additional 50 μL of streptavidin-HRP was added and incubated. Fifty microlitres of DAB substrate was added to the tissue and reacted for 10 min at room temperature. Sections were restained with hematoxylin and sealed by dehydration. Apoptotic cells were photographed and analyzed with ImageJ. A total of 3 random fields per section from 3 individual rats from each group were analyzed. The percentage of TUNEL-positive cells is presented as the number of TUNEL-positive cells/total cell number × 100 (%).

### Quantitative real-time polymerase chain reaction (qRT‒PCR)

MuRF1 and MAFbx are key E3-linked enzymes involved in the ubiquitin protease pathway of muscle degradation. Approximately 50 mg of tibialis anterior from eight rats in each of the four experimental groups was added to 1 mL of TRIzol (G3013, Servicebio, USA) at each time point after injury, and total RNA was extracted according to the manufacturer’s instructions. RNA purity and concentration were measured routinely, complementary DNA was synthesized according to a 20 μL reverse transcription system, and the mRNA expression of MAFbx and MuRF1 was measured by real-time fluorescence quantitative PCR. The upstream primer for MAFbx was 5′-ACATCCCTGAGTGGCATCGC-3′, and the downstream primer was 5′-CATGTTGATGTTGCCCACCA-3′. The upstream primer for MuRF1 was 5′-GTGCCTACTTGCTCCTTGTGC-3′, and the downstream primer was 5′-GGCGTAGAGGGCGTCAAACT-3′. The upstream primer for GAPDH was 5′-CTGGAGAAACCTGCCAAGTATG-3′, and the downstream primer was 5′-GGTGGAAGAATGGGAGTTGCT-3′. Primers were synthesized by Servicebio (Wuhan, China). The mRNA expression of MAFbx and MuRF1 was quantified by the 2^−ΔΔCt^ method based on the ΔΔCycle threshold (Ct), using GAPDH as an internal reference. The mRNA expression in the sham-burn group at 4 days postinjury was used as a reference, and the relative mRNA expression of the remaining groups at various time points was calculated.

### Western blot analysis

Rat TA tissues were lysed and homogenized with radioimmunoprecipitation assay (RIPA) lysis buffer and centrifuged at 12,000×*g* for 20 min at 4 °C. The supernatant was removed, and the protein concentration was measured using a BCA kit. Ten micrograms of protein samples was added to 10% sodium dodecyl sulfate‒polyacrylamide gel electrophoresis (SDS‒PAGE), transferred to nitrocellulose (NC) membranes, and sealed with 5% skim milk for 2 h at room temperature. The membranes were incubated with primary antibodies against muscle atrophy F-box (MAFbx) (Proteintech, 67172-1-1), muscle-specific ring finger protein 1 (MuRF1) (Proteintech, 55456-1-AP) and GAPDH (Servicebio, GB15002), p-IRS1 (CST, 2385), IRS (CST, 2382), p-PI3K (Abcam, ab182651), PI3K (Abcam, ab191606), p-AKT2 (CST, 8599), AKT2 (CST, 3063), p-P70S6K (Abcam, ab59208), P70S6K (Abcam, ab186753), p62 (CST, 39749), LC3 (CST, 4108), GLUT4 (Proteintech, 66846-1-Ig) and β-Actin (TDYbio, TDY051) overnight to wash away the free primary antibody, and then the secondary antibody was incubated for 1 h.

### Metagenomic sequencing and analysis

DNA was obtained using the MgiEasy DNA Stool Kit (BGI, Shenzhen, China) according to the manufacturer’s instructions. DNA library preparation and metagenomic sequencing were performed by BGI, Shenzhen, China. The concentration of all DNA samples was measured using a microplate reader, and agarose gel electrophoresis was used to check sample integrity. Fragments of 200–400 bp were selected after breaking the DNA fragments. Then, the fragment was repaired and amplified by PCR, and purified DNA was heat denatured into single strand cyclized DNA. The concentration was detected, and the library was established by machine sequencing. In this project, a total of 20 samples were tested using the DNBSEQ platform. The software SOAPnuke was used to obtain high-quality clean data, and then Bowtie2 was used to filter out the host sequence. The clean data obtained after fragments below 300 bp were filtered out were then assembled using MEGAHIT, followed by statistical analysis and gene prediction. The average output of each sample was 6.05 G, and the average assembly length was 124.29. The total number of detected gene catalogues was 1,364,314, which were functionally annotated by 7 databases, including BacMet, Card, Cazy, Cog, KEGG, Nog, and SwissProt. The number of species obtained after taxonomic analysis was 4 at the kingdom level, 52 at the phylum level, 99 at the class level, 208 at the order level, 455 at the family level, 1615 at the genus level, and 5727 at the species level.

### Enzyme-linked immunosorbent assay (ELISA) AND glucose oxidase activity assay

Serum samples were collected to measure the levels of glucose using a Glucose Oxidase Activity Assay Kit (NJJCBio, Nanjing, China), and the cytokine levels of TNF-α, IL-6, IL-1β and IL-10 were measured using ELISA kits (Bioswamp, Wuhan, China) according to the manufacturer's instructions.

### Statistical analysis

Statistical analyses were performed using SPSS22.0 and Graph Pad Prism 8.0 software (La Jolla, USA). Measurement data are presented as the means ± standard errors of the means (SEMs). Statistical comparisons among multiple groups were assessed with one-way analysis of variance (ANOVA), while comparisons among groups were analysed by a t test. Differences with* P* < 0.05 were regarded as statistically significant.

### Ethical approval

The animal study protocol was approved by the Animal Ethics Committee of Tongren Hospital of Wuhan University (protocol code WAEF-2022-0157 and date of approval). The study is reported in accordance with ARRIVE guidelines.

### Supplementary Information

Supplementary Figures.

## Data Availability

The data and material are available from the corresponding authors on reasonable request.
