# Supplementary material for: Dietary supplementation with inulin improves burn-induced skeletal muscle atrophy by regulating gut microbiota disorders
Source: Sci Rep. 2024 Jan 28;14:2328. doi: 10.1038/s41598-024-52066-8 (PMC10822858; doi:10.1038/s41598-024-52066-8)

Figure 2b MAFbx-1/ FBXO32


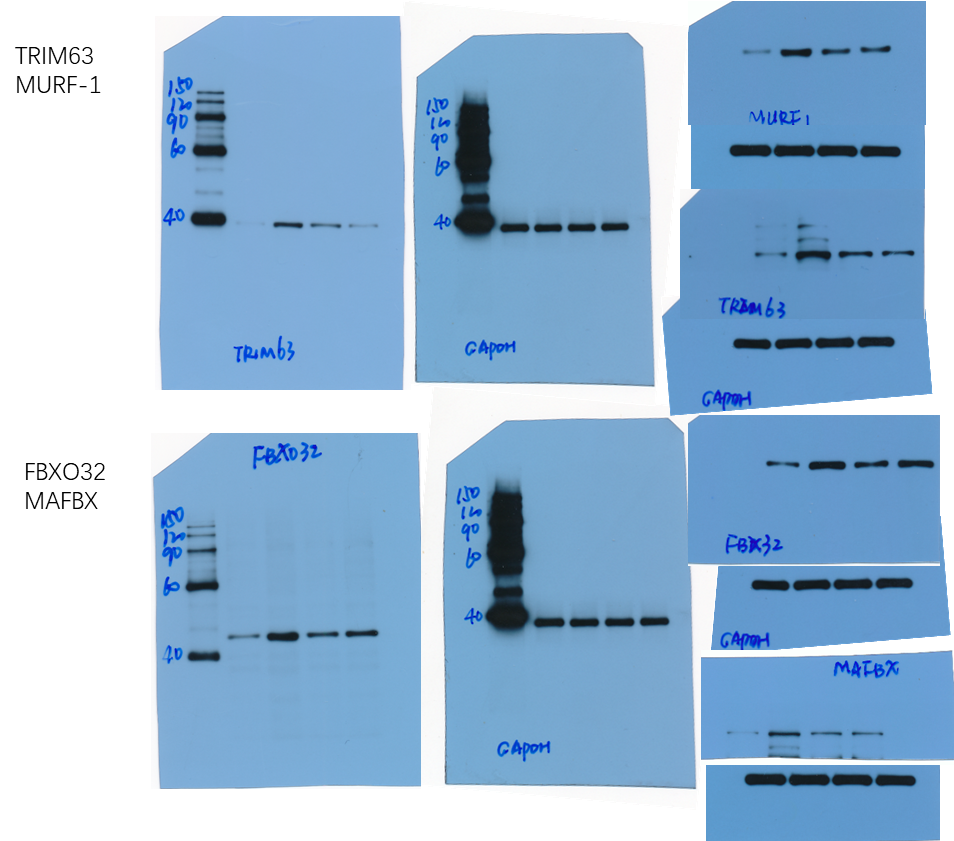


MuRF-1/TRIM63


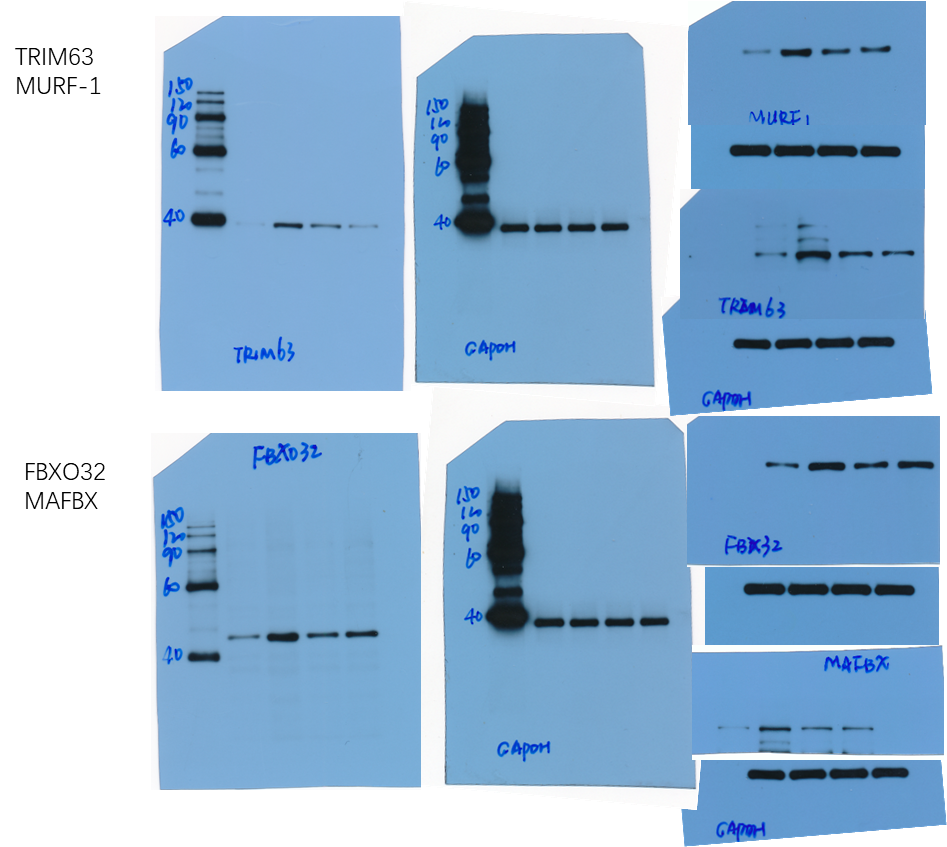


Figure 6a p-IRS1


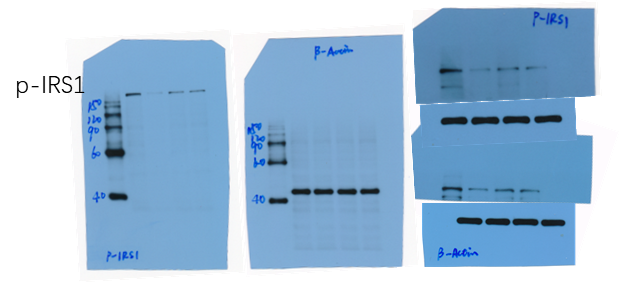


IRS1


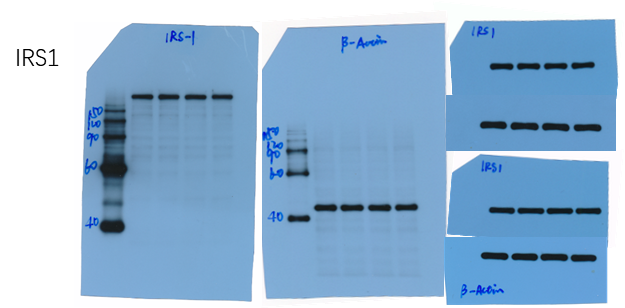


p-PI3K


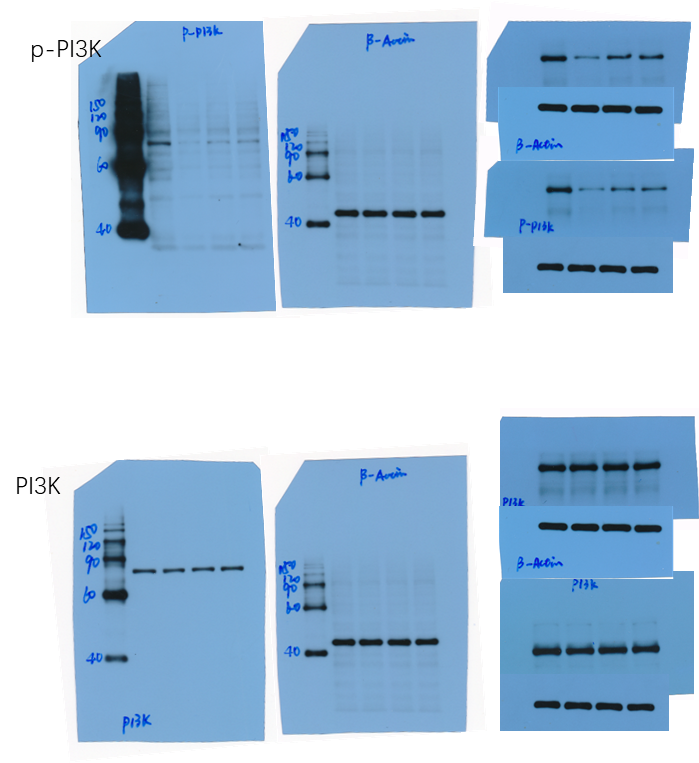


PI3K


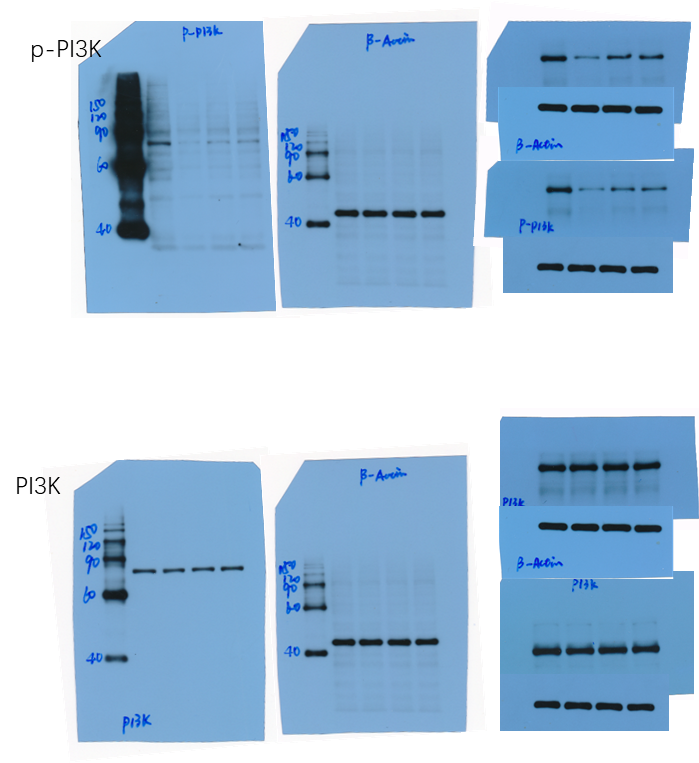


Figure 6b p-AKT2


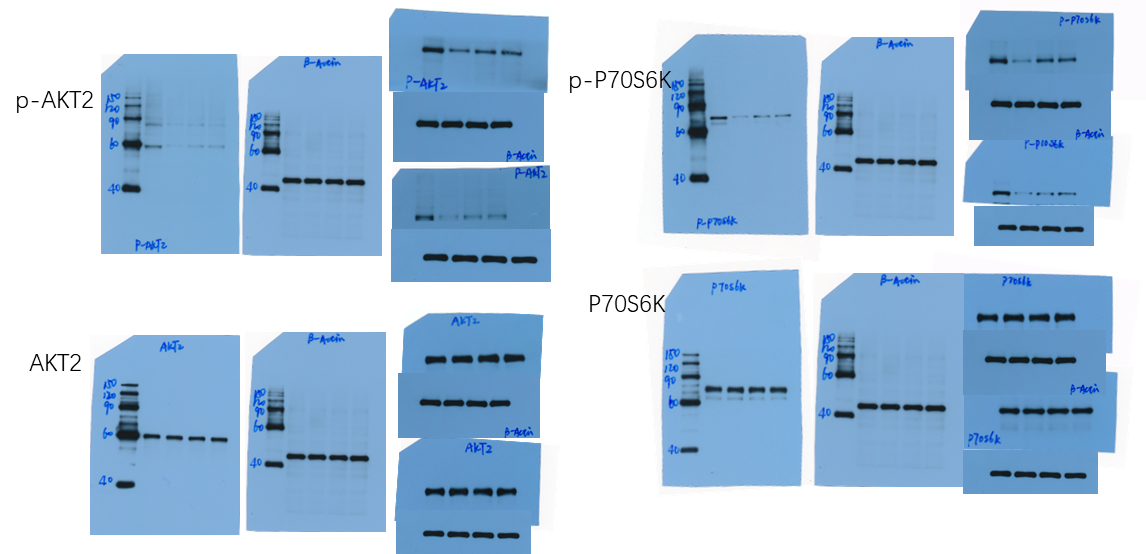


AKT2


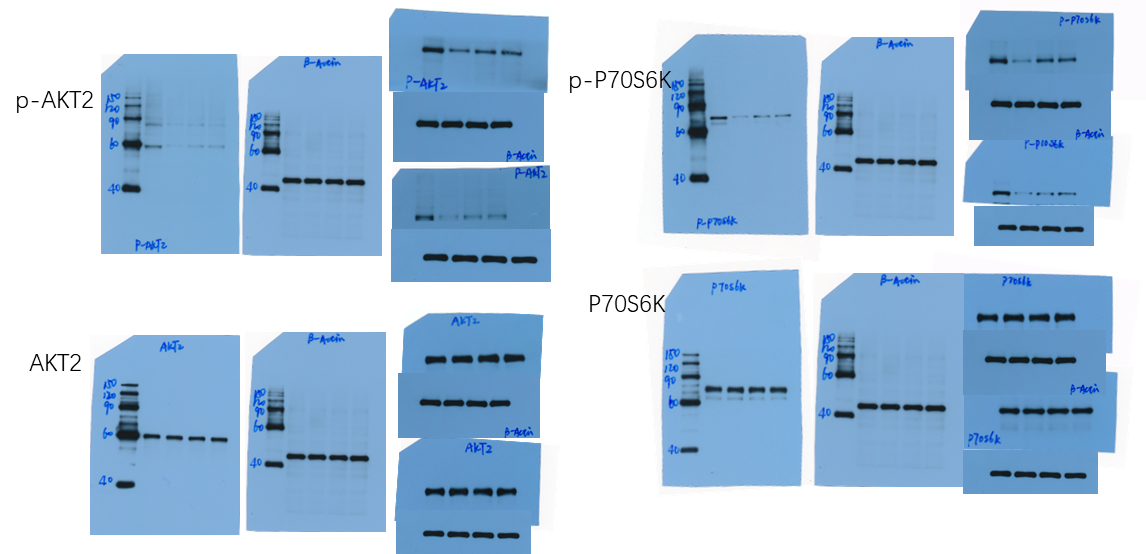


p-P70S6K


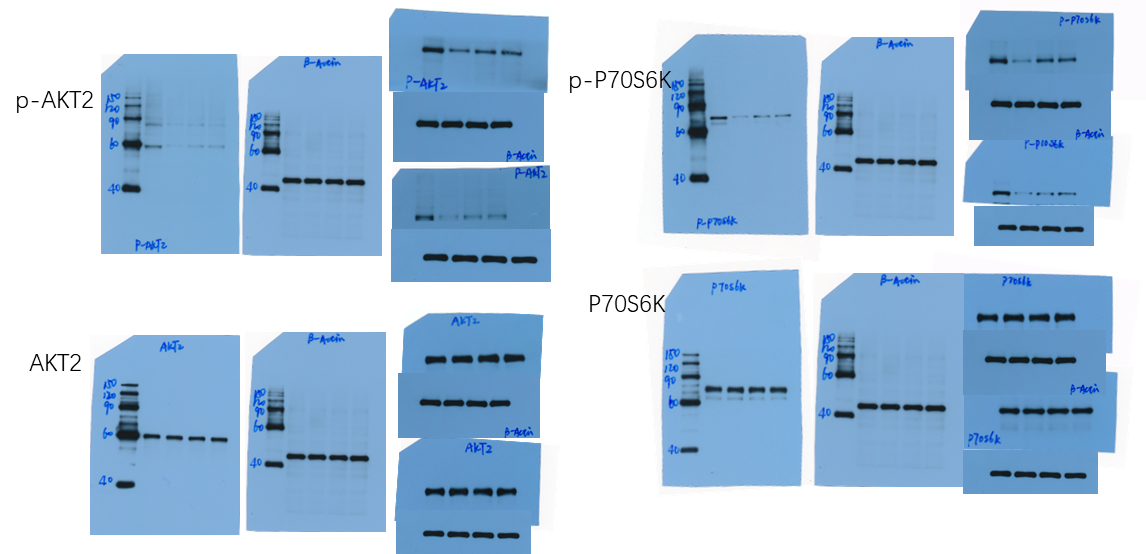


P70S6K


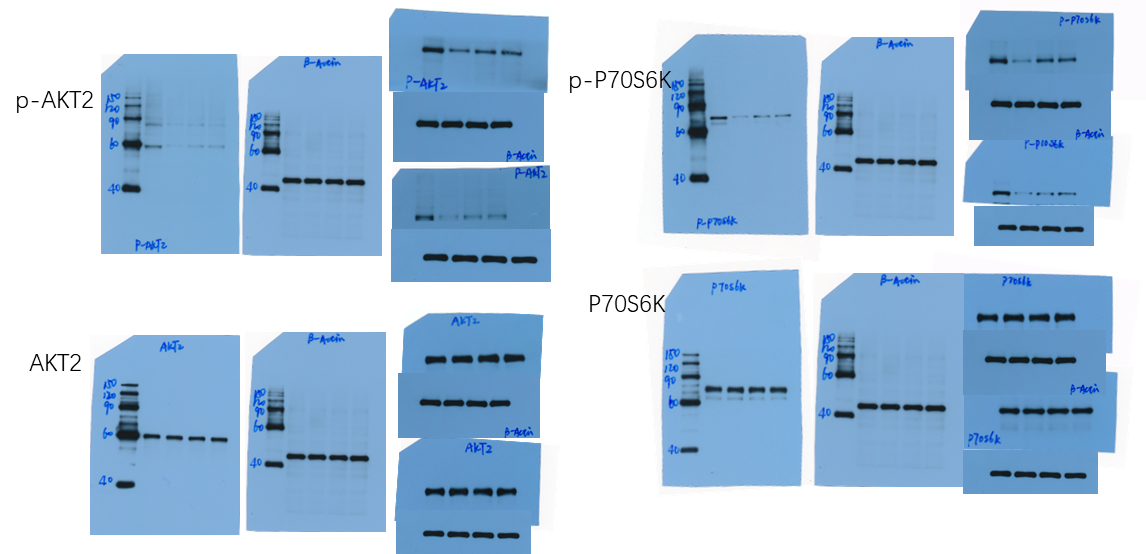


Figure 6c GLUT4


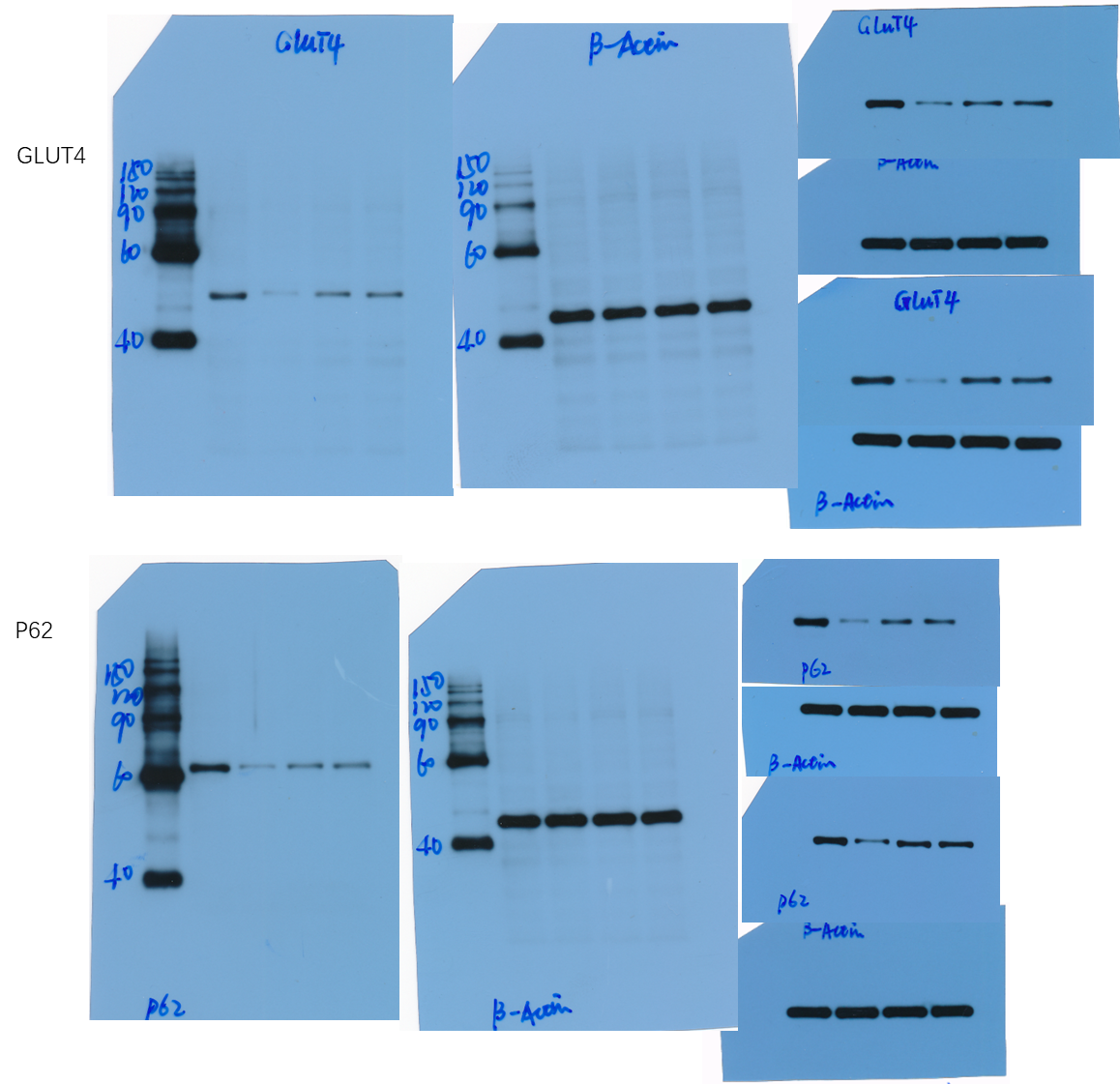


p62


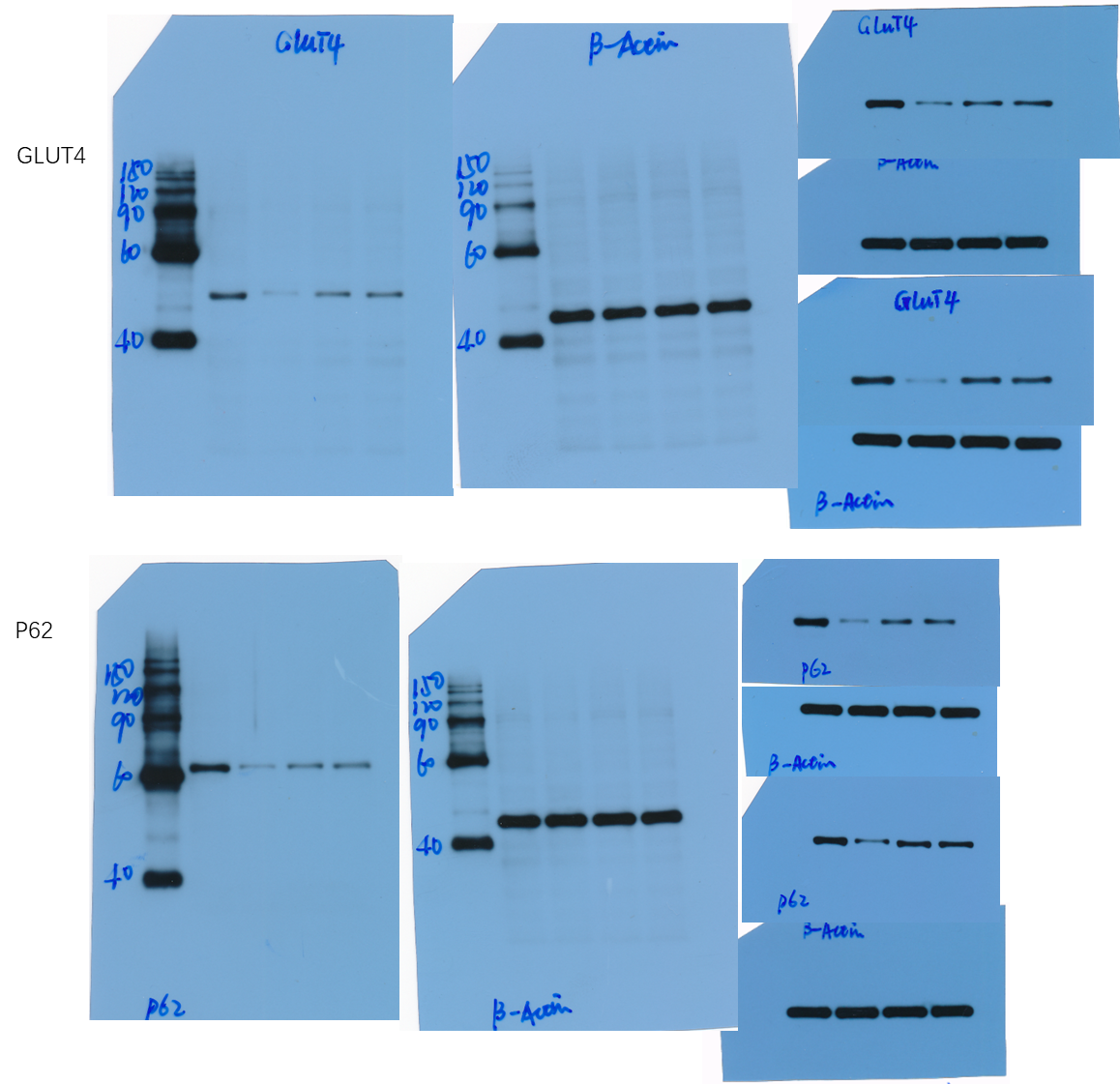


LC3Ⅰ/Ⅱ


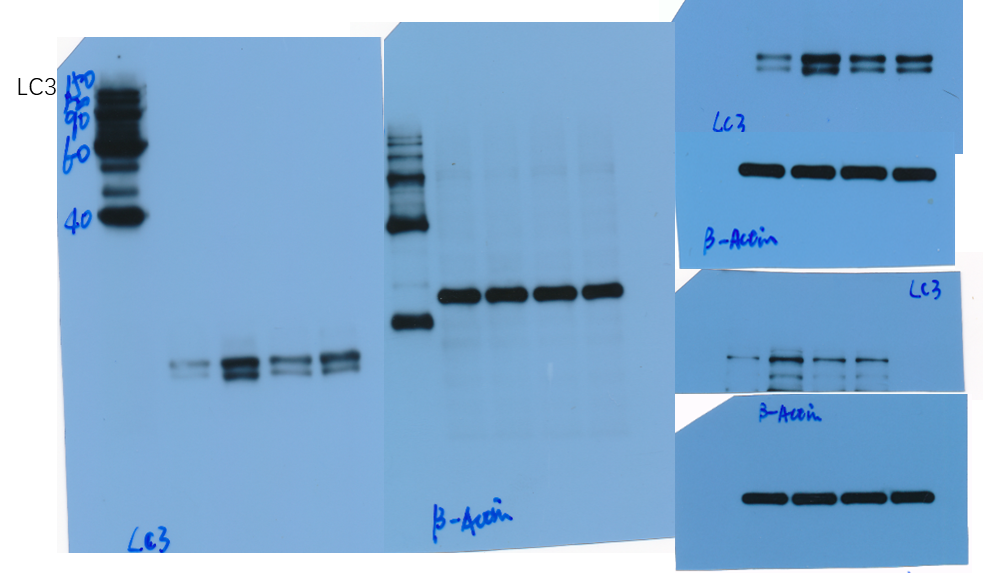

Supplement: Supplementary file 1 — Supplementary Figures. [file 41598_2024_52066_MOESM1_ESM.docx]
